# Temperament in People with Familial High Risk for Bipolar Disorder and Psychosis: An exploration of Schizotaxia and Cyclotaxia

**DOI:** 10.1192/j.eurpsy.2025.584

**Published:** 2025-08-26

**Authors:** M. Demir, M. S. Eyüboğlu, E. Cesim, Ç. Çimentepe-Sezer, B. Yalınçetin, B. Verim, E. T. Süt, E. Bora

**Affiliations:** 1 Department of Neurosciences; 2 Department of Psychiatry; 3 Department of Child and Adolescent Psychiatry, Dokuz Eylül University, İzmir, Türkiye; 4 Department of Psychiatry, University of Melbourne, Melbourne, Australia

## Abstract

**Introduction:**

Research on temperament in individuals with familial high risk for psychosis (FHR-P) and bipolar disorder (FHR-BD) suggests that specific temperament patterns may mirror characteristics of the associated disorders. Terms such as schizotaxia and cyclotaxia describe these patterns: schizotaxia is linked to traits such as cognitive disorganization and social withdrawal seen in those at risk for psychosis, while cyclotaxia involves emotional instability and reactivity associated with bipolar disorder.

**Objectives:**

This study aims to investigate the relationship between temperament traits associated with psychosis and bipolar disorder as suggested in the concept of schizotaxia and cyclotaxia.

**Methods:**

We recruited a total of 94 participants, comprising 29 people at familial high risk for psychosis (FHR-P), 41 people at familial high risk for bipolar disorder (FHR-BD), and 24 controls (HC). Participants from familial high-risk groups were identified through patient records or outpatient settings at Dokuz Eylül University. Several self-report measures are selected to investigate the temperament traits of schizotypy and cyclothymia. Those measures listed as; the Schizotypal Personality Questionnaire (SPQ), Behavioral Inhibition System/Behavioral Activation System (BIS/BAS) Scale, Temperament Evaluation of Memphis, Pisa, Paris and San Diego-auto questionnaire version (TEMPS-A), Pittsburgh Sleep Quality Index (PSQI), Barratt Impulsiveness Scale (BIS). One-way ANOVA is used to analyze the data using Jamovi Version 2.6.2.0.

**Results:**

FHR-P individuals, but not FHR-BD, had significantly higher scores of depressive(p<.001), cyclothymic (p=.005) and anxious(p=.002) temperament than HC in TEMPS-A subscales. Both familial groups had significantly higher scores in activation(p=.001) but not in inhibition in BIS/BAS. Moreover, they had worse sleep quality indicated by significantly higher PSQI (p=.014) scores. BIS scores and SPQ total scores do not significantly differ across groups however, FHR-P had significantly higher scores in negative subscores(p=.032) of SPQ than HC.

**Conclusions:**

Our findings show significant differences in the FHR-P group across most temperament scores, indicating a greater vulnerability to schizotypal and cyclothymic traits. This may result from various factors, such as participants being recruited from outpatient psychiatric settings. Both familial groups also demonstrated lower educational levels and premorbid IQ scores on the WAIS, with more pronounced differences in the FHR-P group compared to the FHR-BD group. Additionally, the unequal sample sizes among the groups could affect the results. Therefore, further research is needed to account for the influence of common psychiatric conditions associated with participants’ help-seeking behaviour and to ensure better educational matching with healthy controls.

**Disclosure of Interest:**

None Declared

